# Droplet-based microfluidic platform for detecting agonistic peptides that are self-secreted by yeast expressing a G-protein-coupled receptor

**DOI:** 10.1186/s12934-024-02379-0

**Published:** 2024-04-09

**Authors:** Ririka Asama, Cher J. S. Liu, Masahiro Tominaga, Yu-Ru Cheng, Yasuyuki Nakamura, Akihiko Kondo, Hsiang-Yu Wang, Jun Ishii

**Affiliations:** 1 Graduate School of Science, Technology, and Innovation, Kobe University, 1-1 Rokkodai, Nada, Kobe, 657-8501 Japan; 2 Department of Engineering and System Science, National Tsing Hua University, Hsinchu, 30013 Taiwan; 3 Engineering Biology Research Center, Kobe University, 1-1 Rokkodai, Nada, Kobe, 657-8501 Japan; 4 Department of Chemical Science and Engineering, Faculty of Engineering, Kobe University, 1-1 Rokkodai, Nada, Kobe, 657-8501 Japan; 5 Center for Sustainable Resource Science, RIKEN, 1-7-22 Suehiro, Tsurumi, Yokohama, 230-0045 Japan; 6 Present Address: Bacchus Bio Innovation Co., Ltd., 6-3-7 Minatojima-Minami, Chuo, Kobe, 650-0047 Japan

**Keywords:** Droplet, G-protein-coupled receptor, Yeast, Microfluidics, Agonistic peptide, Angiotensin, Machine learning

## Abstract

**Background:**

Single-cell droplet microfluidics is an important platform for high-throughput analyses and screening because it provides an independent and compartmentalized microenvironment for reaction or cultivation by coencapsulating individual cells with various molecules in monodisperse microdroplets. In combination with microbial biosensors, this technology becomes a potent tool for the screening of mutant strains. In this study, we demonstrated that a genetically engineered yeast strain that can fluorescently sense agonist ligands via the heterologous expression of a human G-protein-coupled receptor (GPCR) and concurrently secrete candidate peptides is highly compatible with single-cell droplet microfluidic technology for the high-throughput screening of new agonistically active peptides.

**Results:**

The water-in-oil microdroplets were generated using a flow-focusing microfluidic chip to encapsulate engineered yeast cells coexpressing a human GPCR [i.e., angiotensin II receptor type 1 (AGTR1)] and a secretory agonistic peptide [i.e., angiotensin II (Ang II)]. The single yeast cells cultured in the droplets were then observed under a microscope and analyzed using image processing incorporating machine learning techniques. The AGTR1-mediated signal transduction elicited by the self-secreted Ang II peptide was successfully detected via the expression of a fluorescent reporter in single-cell yeast droplet cultures. The system could also distinguish Ang II analog peptides with different agonistic activities. Notably, we further demonstrated that the microenvironment of the single-cell droplet culture enabled the detection of rarely existing positive (Ang II-secreting) yeast cells in the model mixed cell library, whereas the conventional batch-culture environment using a shake flask failed to do so. Thus, our approach provided compartmentalized microculture environments, which can prevent the diffusion, dilution, and cross-contamination of peptides secreted from individual single yeast cells for the easy identification of GPCR agonists.

**Conclusions:**

We established a droplet-based microfluidic platform that integrated an engineered yeast biosensor strain that concurrently expressed GPCR and self-secreted the agonistic peptides. This offers individually isolated microenvironments that allow the culture of single yeast cells secreting these peptides and gaging their signaling activities, for the high-throughput screening of agonistic peptides. Our platform base on yeast GPCR biosensors and droplet microfluidics will be widely applicable to metabolic engineering, environmental engineering, and drug discovery.

**Supplementary Information:**

The online version contains supplementary material available at 10.1186/s12934-024-02379-0.

## Background

Droplet-based microfluidics is a promising technology that permits the manipulation of discrete volumes of fluids in immiscible phases and the encapsulation of cells in monodisperse droplets at the single-cell level [1, 2]. Moreover, it can coencapsulate various molecules, such as compounds and proteins, thus providing a compartmentalized microenvironment for independent reaction or cultivation [3]. Thus, the single-cell droplet microfluidics technology has been increasingly exploited in a variety of biological experiments and is becoming an important platform for high-throughput single-cell analysis and for screening in biomedical, biochemical, and microbial applications [2, 4, 5].

With the advancement of synthetic biology, various microbial biosensors that detect and report analytes have been developed [6]. Some of them have been combined with droplet microfluidics and successfully applied to strain engineering [7–9]. These biosensors are principally used to screen mutant strains that extracellularly produce (secrete) metabolites of interest with high productivity or function at the single-cell level. Droplet microfluidics can avoid issues such as the diffusion and cross-contamination of secreted products [10, 11], as well as population-masking effects for low-fitness phenotypes [12] in the screening using library pools. Thus, single-cell droplets have good compatibility with microbial biosensors because of they offer the advantage of allocating compartmentalized culture environments to each producer [7]. In some cases, each producer of a strain library is coencapsulated and cocultured with different biosensor cells in single droplets [8, 9].

G-protein-coupled receptors (GPCRs), also known as seven-transmembrane receptors [13], are one of the most prominent families used in biosensor fabrication. They respond to diverse external stimuli, including light, small compounds, and peptides, and transduce the signal via coupling with a guanine nucleotide-binding protein (G-protein), to regulate a complex variety of cellular functions [14, 15]. Although mammalian cell lines are the standard choice for GPCR ligand assays, the eukaryotic yeast *Saccharomyces cerevisiae* is an alternate host microorganism for creating simple GPCR biosensors. In addition to its easy genetic modification and fast cell growth, this yeast can also simplify complex signaling pathways because of its monopolistic G-protein, which avoids signaling crosstalk with other G-proteins [16, 17]. Thus,* S. cerevisiae* has been used to engineer in vivo GPCR biosensors, thus affording reporter gene assays for the detection of the activation of G-protein signaling [16]. Based on this information, this yeast has been shown to functionally express over 50 different receptors [16, 18]; furthermore, it has been exploited as a biosensor for the mutational analysis of GPCRs and the drug screening of novel agonistic or antagonistic ligands [19, 20]. More recently, they also have been used as metabolite sensors for metabolic engineering [18, 21].

To screen new ligands using GPCR biosensors, it has been necessary to add their candidates externally to each well in multiwell (e.g., 96-well) plates, to evaluate their agonistic (or antagonistic) activities. However, this approach is labor-intensive and requires further library synthesis. To address this issue, we engineered a GPCR-expressing yeast to artificially self-secrete the peptides as a candidate library and tether them on its cell surface [22, 23]. In principle, the combination of this technique with yeast cell surface display technology [24–27] and the GPCR biosensor allows the concurrent generation and evaluation of library peptides in one cell [22, 23]. Although this method enables the facile cell sorting of each yeast expressing eligible peptides from the mixed culture of the cell library, it requires the fusion between an anchor protein and the peptides, thus raising the concern that steric hindrance of, and/or linker compatibility with the peptides may affect their functionality [28, 29]. To overcome this weakness, Yaginuma et al. coencapsulated peptide-secreting (rather than displaying) yeast cells and GPCR-expressing mammalian reporter cells in a single microdroplet [9]. This report successfully identified the active peptide mutants from the library while highlighting the following future research challenge: the probability of coencapsulation of two different cells in a single droplet is quite low, according to the Poisson distribution [30].

**Fig. 1:**
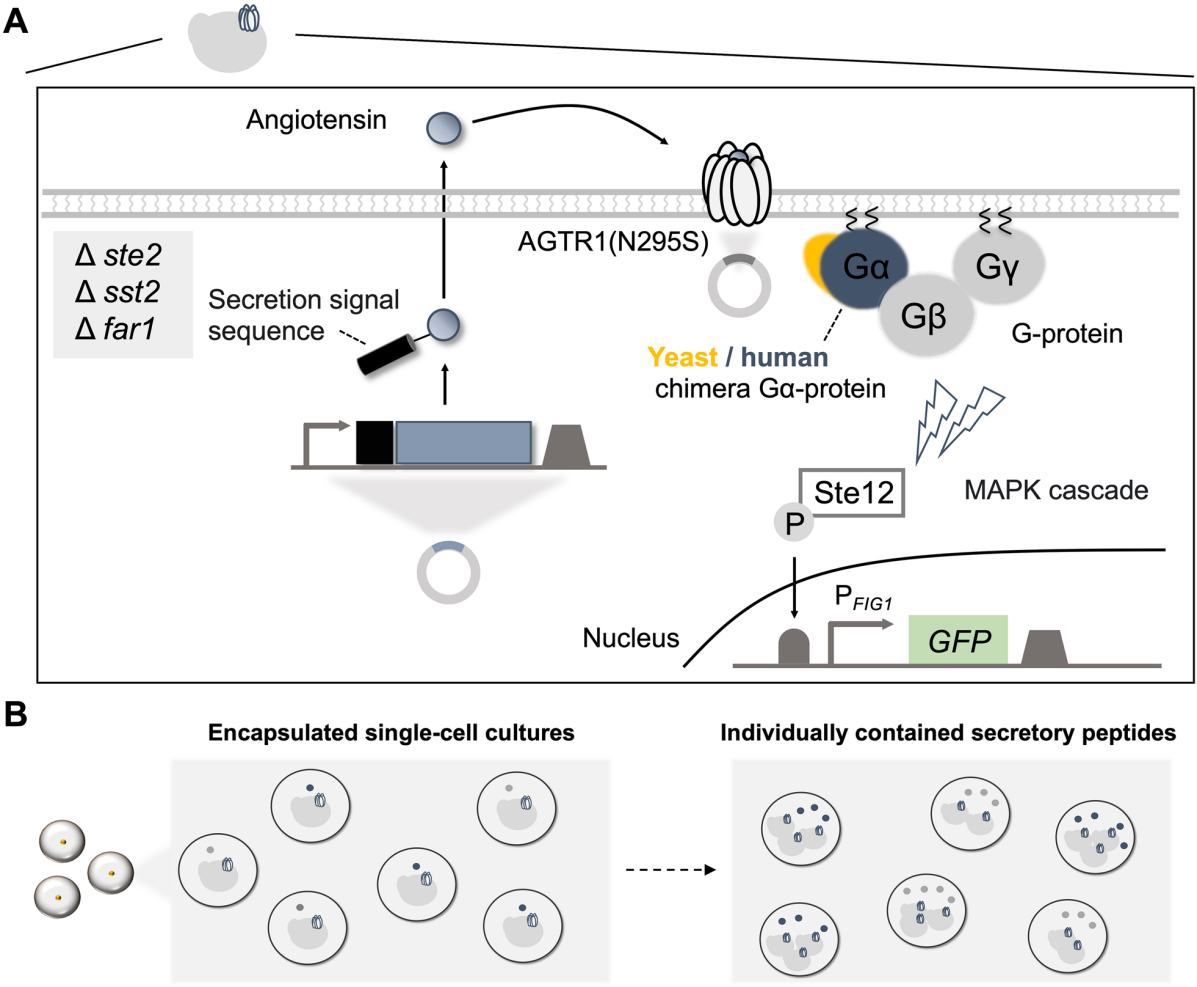
Schematic illustration of the engineered yeast and culture method used in this study. **A** Engineered yeast cells can concurrently express the human AGTR1 receptor (with an N295S mutation) and secrete angiotensin peptides (including analogs). IMFD-72ZsD yeast transformants cointroduced with the GPCR expression plasmid (pGK421-AGTR1-N295S) and the peptide secretion plasmids (pGK426-ssAGII, pGK426-ssAGIII, and pGK426-ssAGIV to secrete the Ang II or its analogs; Ang III and Ang IV) were generated and termed AGII, AGIII, and AGIV, respectively (Table 1). **B** A microdroplet culture in a W/O emulsion is illustrated as the culture method tested in this study

In the current study, we established a new platform to overcome the problems mentioned above by integrating the droplet microfluidics technology into the yeast GPCR biosensor, which was further engineered for secreting (rather than displaying) peptides outside the cells. In this system, single yeast cells were encapsulated in individual droplets, and the produced (different) secretory peptides were encaged within the compartments without diffusing outside of each droplet. Thus, the engineered yeast cells can sense the agonistic activities of the secreted peptides via GPCR by themselves in a unique, independent, and compartmentalized droplet-culture microenvironment, eliminating the need to coencapsulate two different cells (one secreting peptides and the other expressing GPCRs) in a single droplet. Using a yeast strain concurrently expressing the human angiotensin II receptor type 1 (AGTR1, a class-A GPCR) and secreting its cognate ligand (angiotensin II peptide (Ang II)) [31] (Fig. 1A) and water-in-oil (W/O) droplet microfluidics, we demonstrated that our system could distinguish the agonistic activities of the secreted analog peptides (Fig. 1B). Furthermore, we report that the system also provided suitable compartmentalized microculture environments for the single-cell evaluation of rarely existing positive yeast cells in the model library, which may represent a platform for the screening of individual single yeast cells that secrete new agonistic peptides.

**Table 1:** Strains, plasmids, and transformants used in this study

Strain, plasmid, or transformant	Relevant features	References
*Strain*		
BY4741	*MAT***a** *his3*Δ*1 leu2*Δ*0 met15*Δ*0 ura3*Δ*0*	[34]
IMFD-72ZsD	BY4741 *sst2*Δ::*AUR1-C ste2*Δ::*LEU2 fig1*Δ::*ZsGreen his3*Δ::*P*_*FIG1*_*-ZsGreen far1*Δ *gpa1*Δ::*G*_*i3*_*tp*	[22]
*Plasmid*		
pGK421	Yeast expression vector containing the *PGK1* promoter, *PGK1* terminator, 2*μ* origin, and *MET15* marker	[35]
pGK421-AGTR1-N295S	Human AGTR1^N295S^ receptor expression in pGK421	[31]
pGK426	Yeast expression vector comprising the *PGK1* promoter, *PGK1* terminator, 2*μ* origin, and *URA3* marker	[33]
pGK426-ssAGII	Ang II secretory expression in pGK426	[31]
pGK426-ssAGIII	Ang III secretory expression in pGK426	[31]
pGK426-ssAGIV	Ang IV secretory expression in pGK426	[31]
*Transformant*		
AGTR1-N295S	IMFD-72ZsD / pGK421-AGTR1-N295S	[31]
Mock	AGTR1-N295S / pGK426	[31]
AGII	AGTR1-N295S / pGK426-ssAGII	[31]
AGIII	AGTR1-N295S / pGK426-ssAGIII	[31]
AGIV	AGTR1-N295S / pGK426-ssAGIV	[31]

## Methods

### Strains, plasmids, and media

All strains and plasmids to generate transformants used in this study are listed in Table 1. Using the lithium acetate method [32], the *S. cerevisiae* IMFD-72ZsD yeast strain [22] was cotransformed with a receptor expression plasmid (pGK421-AGTR1-N295S [31]) and peptide secretory plasmids (pGK426-ssAGII, pGK426-ssAGIII, or pGK426-ssAGIV [31]). As a control, pGK426 [33] was used for cotransformation, rather than the peptide secretory plasmids.

Synthetic dextrose (SD) medium contained 6.7 g/L of yeast nitrogen base without amino acids (BD Difco, Franklin Lakes, NJ, USA) and 20 g/L glucose was used here. SDM71 medium comprised SD medium containing 200 mM 3-(*N*-morpholino)-2-hydroxypropanesulfonic acid (MOPSO) (Nacalai Tesque, Kyoto, Japan) adjusted to pH 7.1 [31]. Amino acids (20 mg/L histidine and 60 mg/L leucine) were added to the media, to provide the relevant auxotrophic components.

### Batch cultivation for the signaling assay

To evaluate signaling activation, yeast cells were cultivated in batch cultures using shake flasks. The AGTR1 signaling assay for batch cultures was performed basically according to our previously reported procedure [31]. Briefly, the yeast transformants were grown in SD medium in a test tube at 30 °C and 150 rpm overnight, and the cell cultures were then inoculated into 5 mL of SDM71 medium in 10-mL triangular flasks, to afford an initial optical density of 0.1 at 600 nm (OD_600_ = 0.1). The cells were cultured at 30 °C on a rotary shaker at 150 rpm for up to 24 h. The yeast cell cultures were washed and suspended in distilled water for fluorescence microscope observation, or directly diluted with distilled water for flow cytometry analysis.

### Fluorescence microscopy observation

The cells were observed using a BZ-9000 fluorescence microscope (Keyence, Osaka, Japan). Green fluorescence images were acquired using a 470/40 bandpass filter for excitation and a 535/50 bandpass filter for emission.

### Flow cytometry analysis

Fluorescent cells were detected using a flow cytometer (CytoFLEX, Beckman Coulter, Brea, CA, USA) equipped with a 488-nm blue laser and a 525/40 nm bandpass filter. The data were analyzed using the CytExpert 2.0 software (Beckman Coulter). The average green fluorescent protein (GFP) fluorescence (an FITC-A mean) of approximately 10,000 cells was expressed as fluorescence intensity.

### Generation of droplet-encapsulating yeast cells

A flow-focusing microfluidic droplet generator was used to generate droplets encapsulating yeast cells. The aqueous phase containing yeast cells in SDM71 medium (OD_600_ = 0.1) and the oil phase consisting of 3 M™ Novec™ 7500 Engineered Fluid (3 M, St. Paul, MN, USA) with 2% dSURF (Flugient, Le Kremlin-Bicêtre, France) were separately loaded into Eppendorf tubes. The tubes were connected to the microfluidic chip inlets using small-bore PEEK tubing (1/32″ OD × 0.01 mm ID) and controlled by microfluidic flow controllers (Flow EZ™, Fluigent). Once stable-sized droplets with a diameter of approximately 80 μm were formed, they were transferred from the microfluidic chip outlet into the Eppendorf tubes through PEEK tubing. The process continued until a total volume of 100 μL of yeast-encapsulated droplets had been collected.

### Droplet cultivation

The Eppendorf tubes containing droplets were covered with parafilm, to maintain the controlled size of the droplets and provide an isolated environment for the yeast cells, then incubated at 30 °C for static culture. At specific time points (3, 6, and 9 h after the initiation of cultivation), droplets were collected to assess the fluorescence intensity of the yeast cells. The fluorescence emitted by the yeast cells within the droplets was examined and measured using the fluorescence-detection method described below.

### Machine learning image processing for fluorescent yeast cells in droplets

To assess the signaling activation of GFP expression by human AGTR1, yeast cells encapsulated within droplets were cultured at 30 °C for the designated time intervals. After incubation, the droplets containing yeast cells were observed using a fluorescence microscope (BZ-9000, Keyence). Digital images of the yeast cells, specifically those exhibiting green fluorescence, were subjected to analysis using the Fiji software (NIH, Bethesda, MD, USA) and a custom macro developed in-house. The custom macro used in this study incorporated the Weka segmentation plugin, which combines a machine learning algorithm (default setting of the Random Forest algorithm) and selected image features (including Gaussian blur, Sobel filters, difference of Gaussians, Hessian matrix eigenvalues, and membrane projections), for accurate droplet identification and image segmentation. After the application of this plugin to classify the droplets containing cells, a second macro was used to save the detected droplets as a single image and measure the average cell brightness in each droplet in the green channel (expressed as a grayscale value).

## Results and discussion

### Features on the engineered yeast biosensor expressing a GPCR and concurrently secreting an agonistic peptide

To prepare the yeast transformant AGTR1-N295S as the GPCR biosensor, we used the previously developed IMFD-72ZsD yeast strain [22] and pGK421-AGTR1-N295S plasmid [31] (Table 1). Briefly, the IMFD-72ZsD strain carries three gene deletions (Δ*ste2*, Δ*sst2*, and Δ*far1*) and exhibits the following properties: avoiding the competitive expression of endogenous yeast GPCR [35–38]; improving ligand sensitivity; and allowing cell growth and plasmid retention, even during the signal-activated states [36, 39, 40] (Fig. 1A). Furthermore, the yeast Gα-subunit (Gpa1p) was replaced with a yeast-human chimeric Gα (Gpa1p/Gα_i3_ transplant; G_i3_tp), to permit efficient coupling with the Gα_i_-dependent GPCRs; in contrast, two copies of a *GFP* reporter gene (*ZsGreen*) were chromosomally integrated for expression under the control of the signal-responsive *FIG1* promoter (P_*FIG1*_), for the efficient fluorescence-based detection of agonist-stimulated signaling [35, 37, 41] in the IMFD-72ZsD strain (Fig. 1A). The pGK421-AGTR1-N295S plasmid expresses the Gα_i_-dependent AGTR1 receptor with a mutation to a serine residue at Asn295, which is known to increase the binding affinities for angiotensin peptides [31] (Fig. 1A).

Using AGTR1-N295S as the parental strain, the yeast was further transformed with the plasmids to secrete the angiotensin peptides (pGK426-ssAGII, pGK426-ssAGIII, and pGK426-ssAGIV) or the mock plasmid (pGK426) (Table 1). Because the resultant transformants (AGII, AGIII, and AGIV) secreted Ang II or its analogs (Ang III and Ang IV) outside the cells, the secreted peptides bound to the AGTR1 receptor and activated the intracellular mitogen-activated protein kinase cascade via the G-protein, thus inducing the generation of GFP fluorescence signals as a reporter in the cells (Fig. 1A).

### Features of droplet-based single-cell microfluidics to detect GPCR agonists using the engineered yeasts

To validate whether the droplet-based microfluidics can provide compartmentalized single-cell culture microenvironments that are suitable for sensing the agonistic activities of the peptides secreted from the engineered yeasts, we tested a “droplet culture” method that encapsulated the single yeast cells using W/O emulsions (Fig. 1B). A “batch culture” method using a small shake flask was also tested for comparison (Additional file 1: Fig. S1). The latter method (batch culture) diffused the secreted peptides into the medium, rendering it difficult to distinguish the transformant that secreted superior peptides if the cell library was cultured in a mixed state. Therefore, each colony must be inoculated separately into multi-well plates (or flasks), to carry out the screening. In contrast, the former method (droplet culture) allows the individual containment of single yeast cells in the separate droplets, thus providing an independent culture microenvironment for each transformant. Thus, the microdroplet culture can avoid the cross-contamination of each peptide secreted from the different cells, with a potential to enable the high-throughput screening of a strain library that contains the evolved secretory peptides.

**Fig. 2:**
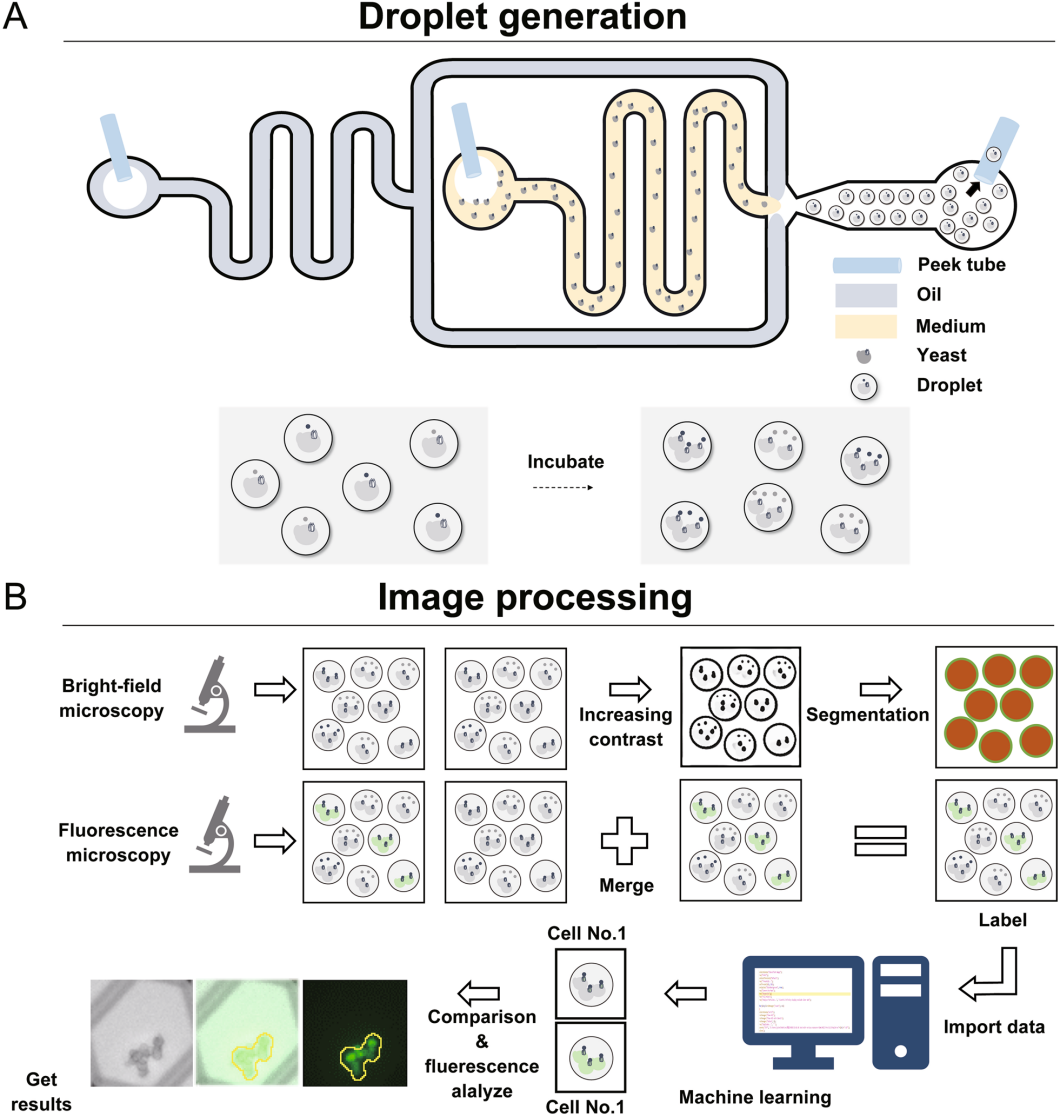
Microfluidics-based droplet generation and image processing for the evaluation of the fluorescence intensity of single yeast cells in microdroplets. The workflows pertain to the production of droplets encapsulating single yeast cells and to the microscopic image-processing method using machine learning. **A** The process of droplet generation using a flow-focusing microfluidic chip. **B** Image-processing method incorporating machine learning techniques for cell observation and evaluation of the fluorescence intensity

Here, we describe a two-step workflow that was developed for the droplet-based fluorescent evaluation of the GPCR agonists using the engineered yeasts; the first half provided droplet generation to encapsulate the single yeast cells (Fig. 2A), whereas the second half consisted in image processing using machine learning to observe cells in the droplet and evaluate their fluorescence intensities based on microscopic images (Fig. 2B).

First, a flow-focusing microfluidic droplet generator was employed to create droplets that encapsulated single yeast cells (Fig. 2A). The yeast cell suspension and oil phase were separately loaded into the device. The control of the flow rates of the two phases enabled the formation of consistent-size droplets that encapsulated single yeast cells. The droplets were then collected and maintained in an incubator at 30 °C for cell growth. For image processing, we observed droplet samples under a fluorescence microscope at specific time points (3, 6, and 9 h), and used the Fiji software and a custom macro incorporating machine learning techniques (Fig. 2B). The macro employed the Weka segmentation plugin to identify droplets with cells, performed yeast cell classification in bright-field images, and measured the average brightness from the green channel (Additional file 1: Fig. S2). Thus, we successfully monitored the fluorescence emitted by single yeast cells in droplets, which contributed to the advancement of the high-throughput analysis and characterization of agonistic peptides against GPCRs.

### Droplet-based detection of AGTR1 signaling stimulated by angiotensin peptides secreted from single yeast cells

**Fig. 3:**
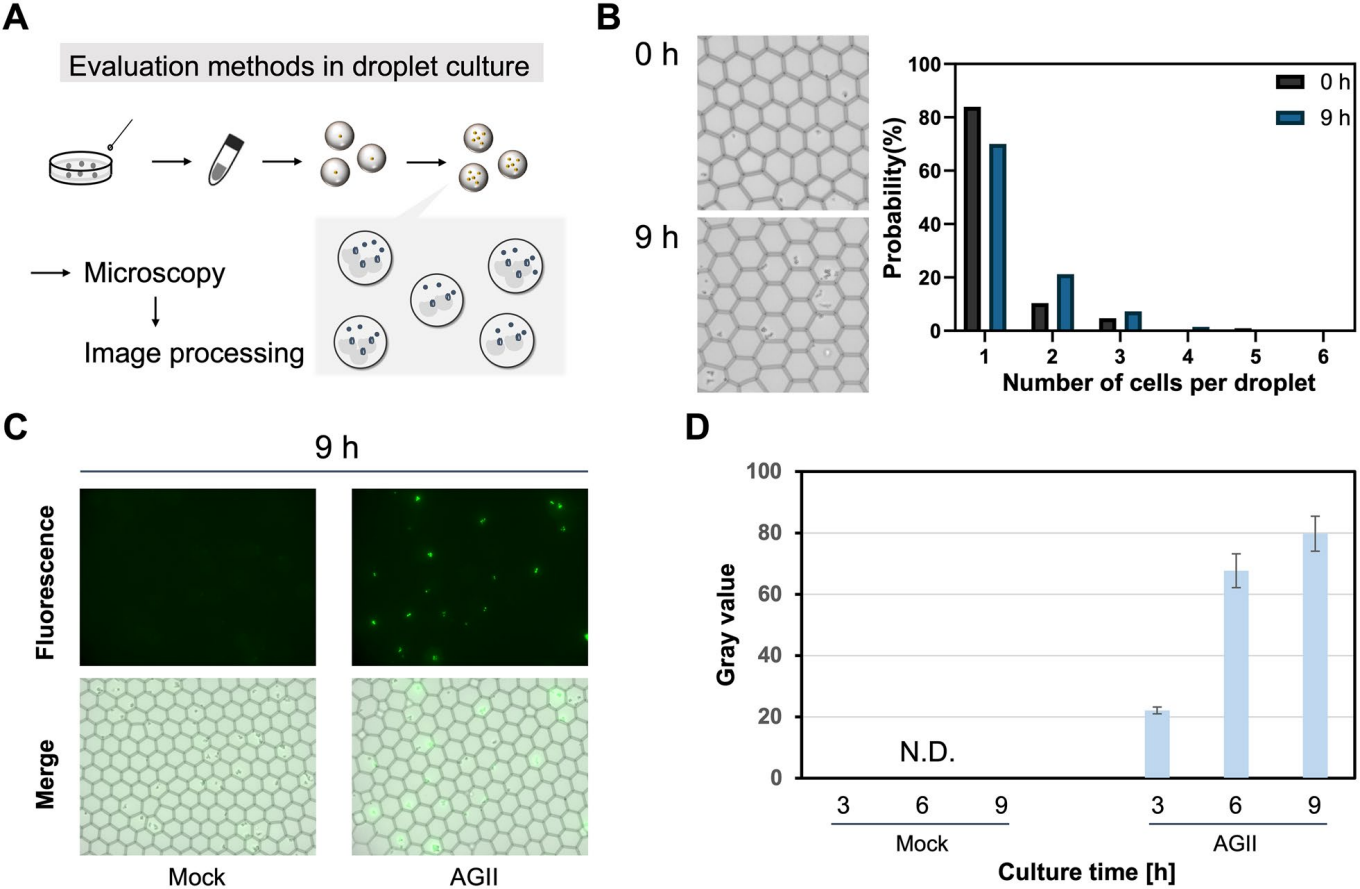
Fluorescence-based detection of the AGTR1 signaling stimulated by the secreted Ang II peptide using engineered yeast in droplet cultures. **A** Single colonies were picked up and cultured in SD medium in test tubes. W/O droplets were generated to encapsulate single yeast cells in SDM71 medium, as shown in Fig. 2A, and then statically incubated at 30 °C. The yeast cells in droplets were observed under a fluorescence microscope, and the gray values were measured by machine-learning-based image processing, as shown in Fig. 2B. **B** Bright-field images of droplets incorporating Ang II-secreting yeast cells (AGII), and the percentage of droplets incorporating the different yeast cell counts (excluding empty droplets). **C** Fluorescence images of yeast-incorporated droplets in droplet cultures at 9 h of cultivation. **D** Time course of the GFP gray values of the yeast cells contained in the P2′ region (Additional file 1: Fig. S4B; fluorescent population) under droplet-culture conditions. The gray values of > 100 samples of yeast-incorporated droplets were calculated. The error bars represent the mean (± SD) of more than 100 samples. N.D. = not detected

First, we examined whether the AGTR1-mediated signaling response could be triggered by the Ang II peptide secreted from yeasts, in batch (Additional file 1: Fig. S3A) and droplet (Fig. 3A) cultures. A peptide-non-secreting (non-expressing) yeast strain (mock) was used as the negative control. Preliminarily, we analyzed the cell population of the AGII yeast cells on the flow cytometer, which confirmed that a certain proportion of cells did not exhibit fluorescence (Additional file 1: Fig. S4A). Because the problem of the loss of the autonomously replicating 2*μ* plasmid is sometimes discussed, even under selective pressure [33, 42], and its activation reduces the plasmid-retention rate [43], the engineered yeast cells that were cotransformed with two pieces of the 2*μ* plasmids must have presented some degree of plasmid loss in the population. Thus, the gating strategy was employed to exclude such non-fluorescent cells, thereby including only the fluorescent cells; moreover, the P2 region was set as the gate in a histogram plot of flow cytometry to measure the average GFP fluorescence intensity in the yeast cells (P2% total of the mock cells < 1%) (Additional file 1: Fig. S4A).

As reported previously [31], the Ang II-dependent GFP expression in the AGII yeast in batch cultures was clearly observed using both fluorescence microscopy (Additional file 1: Fig. S3B) and flow cytometry (Additional file 1: Fig. S3C). The fluorescence signal increased with the incubation time, up to 12 h, but then decreased, probably because of the desensitization of the signaling. After 9 h of incubation, the AGII yeast still exhibited a fluorescence intensity that was more than 20-fold higher than that of the mock cells (Additional file 1: Fig. S3C).

Similarly, droplet cultures were also performed up to 9 h. As in the case of the common single-cell analysis, we first confirmed that > 80% of the droplets, except for the empty ones, encapsulated single yeast cells before cultivation (Fig. 3B and Additional file 1: Fig. S5). If empty droplets were included, approximately 20% of the droplets encapsulated single yeast cells (Additional file 1: Fig. S5), indicating a higher probability than that of the coencapsulation of two different cells (0.74%) [30]. Among all yeast cells encapsulated in the droplets, 14% were moderately dividing in the W/O microdroplets after 9 h of incubation (Fig. 3B). Similar to that observed in the flow cytometry experiment, the gating strategy was used to exclude the non-fluorescent cells and include the fluorescent cells, and the P2′ region was set as the gate in a histogram plot of machine-learning-based image processing, to measure the average GFP brightness (gray value) of the yeast cells that were encapsulated in the droplets (P2′% total of the mock cells < 1%) (Additional file 1: Fig. S4B). At 9 h of incubation in droplets in the medium, the Ang II-secreting yeast cells provided clear fluorescence images (Fig. 3C) and showed higher gray values (Fig. 3D). Therefore, the yeast cells that concurrently expressed the AGTR1 receptor and secreted Ang II were able to stimulate GPCR signaling, even in the compartmentalized microculture environment within the W/O microdroplet, as observed in the batch cultures.

### Droplet-based measurement of the agonistic activity of angiotensin analog peptides

**Fig. 4:**
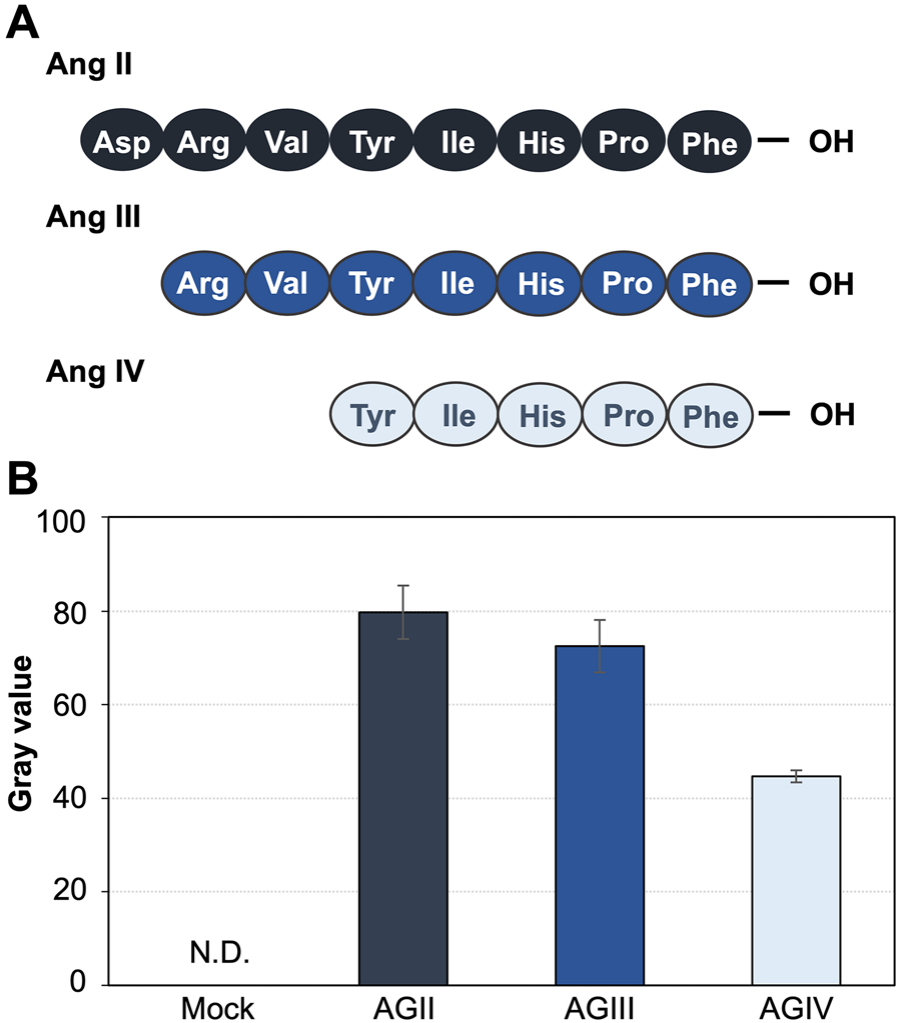
Fluorescence-based detection of AGTR1 signaling stimulated by the secreted angiotensin analog peptides using engineered yeasts in droplet cultures. **A** Amino acid sequences of the Ang II peptide and its analogs. **B** GFP gray values of yeast cells contained in the P2′ region (Additional file 1: Fig. S4B) in droplet cultures at 9 h of incubation. The error bars represent the mean ± SD of more than 100 samples. Cultivation and analyses were performed using the same procedures as those described in Fig. 3. N.D. = not detected

After the establishment of the droplet-based system to evaluate AGTR1-mediated signaling in yeast, we next tested if the system was able to distinguish the agonistic activities of different ligands. Ang II analogs with lower activities, i.e., the heptapeptide Ang III [44] and the hexapeptide Ang IV [45] (Fig. 4A), were used both for droplet and batch cultures. As expected, the yeast cells exhibited a weaker fluorescence both in droplet and batch cultures as the agonistic activities of the angiotensin peptides decreased (Fig. 4B and Additional file 1: Fig. S6). The relative responsiveness to these three peptides remained largely unchanged between the droplet and batch cultures. Thus, we confirmed that our approach could evaluate the agonistic activity of the GPCR analog peptides, even in the compartmentalized microenvironmental cultures of droplets encapsulating single yeast cells.

### Droplet-based discrimination of single-cell yeasts that secreted GPCR agonistic peptides from a mixed library

**Fig. 5:**
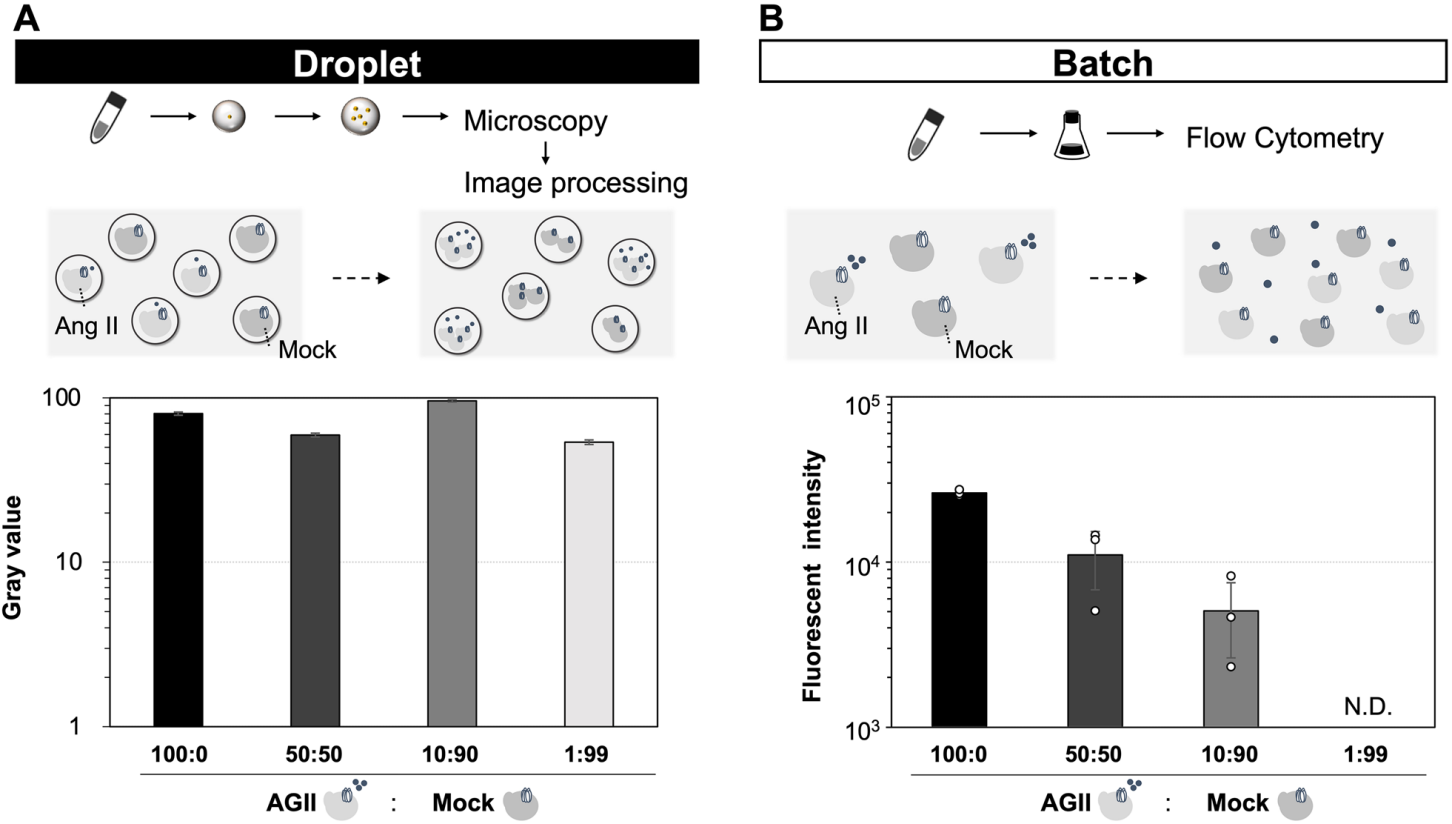
Detection of fluorescence among mixed yeast cells in batch and droplet cultures. Model yeast libraries were prepared using different mixing ratios of Ang II-secreting yeast cells (AGII) and non-secreting yeast cells (mock). **A** GFP gray values of the yeast cells contained in the P2′ region (Additional file 1: Fig. S4B) in droplet cultures. Single colonies of the AGII and mock strains were picked up and cultured separately in SD medium in test tubes. Using the mixed cell cultures with the indicated ratios, W/O droplets were generated to encapsulate single yeast cells in SDM71 medium, as shown in Fig. 2A, and then statically incubated at 30 °C for 9 h. The yeast cells in the droplets were observed under a fluorescence microscope, and the gray value was measured by machine-learning-based image processing, as shown in Fig. 2B. The gray values of > 100 samples of yeast-incorporating droplets were calculated, and the average gray values of yeast cells in the P2′ region were extracted. The error bars represent the mean ± SD of more than 100 samples. **B** GFP fluorescence intensities in the P2 region (Additional file 1: Fig. S4A) in batch cultures. Single colonies of the AGII and mock strains were picked up and cultured separately in SD medium in test tubes. The mixed cell cultures with the indicated ratios were inoculated into SDM71 medium in flasks, and then cultured at 30 °C, and shaken at 150 rpm for 9 h. Approximately 10,000 cells were analyzed by flow cytometry, and the average fluorescence intensities of yeast cells in the P2 region were calculated. The error bars represent the mean ± SD of three independent experiments. N.D. = not detected

Finally, we tested whether our approach had the potential future application of screening of single yeast cells that secreted the promising candidates of agonistic peptides. To verify this hypothesis, model yeast libraries with different mixing ratios of Ang II-secreting cells (AGII) and non-secreting cells (mock) were prepared (Fig. 5). These libraries were subsequently subjected to droplet and batch cultures, followed by analysis using microscopy-based image processing and flow cytometry, respectively (Fig. 5A and B).

In the droplet-culture environments, the average GFP gray values of fluorescent yeast cells were roughly similar among all of the cell-mixing ratios tested (Fig. 5A). In contrast, in the batch-culture environments, the average GFP fluorescence intensities of fluorescent yeast cells decreased gradually as the mixing ratio of Ang II-secreting cells decreased (Fig. 5B). At a ratio of Ang II-secreting cells to mock cells of 1:99, no fluorescent cells could be detected, even though approximately 10,000 yeast cells were analyzed via flow cytometry (Fig. 5B).

These results clearly demonstrated that the secreted peptides diffused in the batch-culture environments and became diluted in the culture medium, whereas the droplet cultures successfully provided the compartmentalized microenvironments that are necessary to culture the single yeast cells and contain the secreted peptides within the microdroplets. However, our approach for evaluating and isolating droplets still faces certain challenges, especially when scaling up the evaluation process. To minimize signal variation, uniform incubation durations of the huge number of droplets before detection are necessary. Moreover, it would be preferable to incubate the droplets using a continuous flow device instead of a batch container to ensure homogeneous oxygen availability [46]. For detecting and separating single yeast cells, our approach can be combined with fluorescent image-based sorting technologies that use image processing and dielectrophoretic or microvalve sorting [47–50]. This will enable us to screen yeast cells that secrete active agonistic peptides in the future.

## Conclusions

In this study, we demonstrated that the integration of the droplet-based microfluidics technology with a yeast-based GPCR biosensor that self-secreted the peptides allowed the single-cell evaluation of the agonistic activity of the peptides secreted by the yeast in individual microdroplets. In our platform, the engineered yeast biosensor strain concomitantly expressed the GPCR and secreted the peptide in one cell, and was analyzed using the single-cell droplet microfluidic technology incorporating machine-learning-based image-processing techniques. The encapsulated single yeast cells could produce the secretory peptides and further sense their agonistic activities within the individual droplets separately, thus providing unique, independent, and compartmentalized microculture environments. Because this system circumvented the drawbacks of the process, including the diffusion, dilution, and cross-contamination of the secreted peptides in library pools, the scaling up of our microdroplet evaluation method in combination with other image-based sorting technologies would enable the high-throughput screening of new agonistically active peptides from large-scale libraries. Because of the wide repertories of GPCRs that have been assorted to date, our approach will be applicable to the fields of metabolic engineering, environmental engineering, and drug discovery.

### Supplementary Information

**Additional file 1: Figure S1.** Schematic illustration of the batch culture method in a small shake flask used in this study. **Figure S2.** Custom macro incorporating machine learning techniques to identify droplets encapsulating yeast cells. We used the “Trainable Weka segmentation” of the Fiji software for image processing in two steps: automatic single droplet image extraction and individual cell classification from the droplets. The learning method involved training the algorithm to recognize cell and droplet features to complete these steps. This approach is crucial for efficient data analysis, ensuring accuracy and speed. **Figure S3.** Fluorescence-based, secreted Ang II peptide-stimulated AGTR1 signaling detection using engineered yeast in batch cultures. (A) Single colonies were picked up and cultured in SD medium in test tubes. The cell cultures were then inoculated into SDM71 medium in flasks, and cultured at 30°C, shaken at 150 rpm. We observed the yeast cells under a fluorescence microscope and measured the fluorescence intensity using a flow cytometer. (B) Fluorescence images of yeast cells in batch cultures at 9 h of culture. (C) Time course of GFP fluorescence intensities in the P2 region of yeast cells (Additional file 1: Fig. S4A; fluorescent population) under batch-culture conditions. Approximately 10,000 cells were analyzed using flow cytometry. The error bars represent the mean ± SD of three independent experiments. N.D. = not detected. **Figure S4.** Setting of the P2 and P2′ regions in batch and droplet cultures, respectively. The engineered yeast cells (Mock and Ang II; Table 1) were used to determine the gate regions. (A) Histogram plots of the GFP fluorescence intensities of yeast cells cultured in batch flasks. Three independent yeast colonies were grown in flasks for 9 h, and approximately 10,000 cells were analyzed by flow cytometry. The P2 region was set to exclude non-fluorescent cells and include only fluorescent cells in the histogram plots of flow cytometry (P2% total in mock cells <1%). (B) Histogram plots of the GFP gray values of yeast cells cultured in single-cell microdroplets. The yeast cells were grown in W/O droplets for 9 h and observed under a fluorescence microscope. The gray values of >100 samples of yeast-incorporating droplets were measured by machine-learning-based image processing, as shown in Fig. 2B. The P2′ region was set to exclude non-fluorescent cells and include only fluorescent cells in the histogram plot of microscopy-based image processing (P2′% total in mock cells <1%). **Figure S5.** Number of yeast cells encapsulated in a single droplet before cultivation (including empty droplets). **Figure S6.** Fluorescence-based detection of secreted angiotensin analog peptide-stimulated AGTR1 signaling using engineered yeasts in batch cultures. GFP fluorescence intensities in the P2 region of yeast cells (Fig. S4A) in batch cultures at 9 h of culture. The error bars represent the mean ± SD of three independent experiments. Culture and analyses were performed using the procedures described in Fig. S3. N.D. = not detected.

## Data Availability

The datasets supporting the conclusions of this article are included in the manuscript and additional files.
