# Supplementary material for: Droplet-based microfluidic platform for detecting agonistic peptides that are self-secreted by yeast expressing a G-protein-coupled receptor
Source: Microb Cell Fact. 2024 Apr 9;23:104. doi: 10.1186/s12934-024-02379-0 (PMC11005146; doi:10.1186/s12934-024-02379-0)
Supplement: Supplementary file 1 — Additional file 1: Figure S1. Schematic illustration of the batch culture method in a small shake flask used in this study. Figure S2. Custom macro incorporating machine learning techniques to identify droplets encapsulating yeast cells. We used the “Trainable Weka segmentation” of the Fiji software for image processing in two steps: automatic single droplet image extraction and individual cell classification from the droplets. The learning method involved training the algorithm to recognize cell and droplet features to complete these steps. This approach is crucial for efficient data analysis, ensuring accuracy and speed. Figure S3. Fluorescence-based, secreted Ang II peptide-stimulated AGTR1 signaling detection using engineered yeast in batch cultures. (A) Single colonies were picked up and cultured in SD medium in test tubes. The cell cultures were then inoculated into SDM71 medium in flasks, and cultured at 30°C, shaken at 150 rpm. We observed the yeast cells under a fluorescence microscope and measured the fluorescence intensity using a flow cytometer. (B) Fluorescence images of yeast cells in batch cultures at 9 h of culture. (C) Time course of GFP fluorescence intensities in the P2 region of yeast cells (Additional file 1: Fig. S4A; fluorescent population) under batch-culture conditions. Approximately 10,000 cells were analyzed using flow cytometry. The error bars represent the mean ± SD of three independent experiments. N.D. = not detected. Figure S4. Setting of the P2 and P2′ regions in batch and droplet cultures, respectively. The engineered yeast cells (Mock and Ang II; Table 1) were used to determine the gate regions. (A) Histogram plots of the GFP fluorescence intensities of yeast cells cultured in batch flasks. Three independent yeast colonies were grown in flasks for 9 h, and approximately 10,000 cells were analyzed by flow cytometry. The P2 region was set to exclude non-fluorescent cells and include only fluorescent cells in the histogram plots of fl [file 12934_2024_2379_MOESM1_ESM.docx]

**Additional File 1 (Supplementary Figures)**

**Droplet-based microfluidic platform for detecting agonistic peptides that are self-secreted by yeast expressing a G-protein-coupled receptor**

**Ririka Asama^1†^, Cher J.S. Liu^2†^, Masahiro Tominaga^1,3^, Yu-Ru Cheng^2^, Yasuyuki Nakamura^1,3,§^, Akihiko Kondo^1,3,4,5^, Hsiang-Yu Wang^2^*, Jun Ishii^1,3^***

1 Graduate School of Science, Technology, and Innovation, Kobe University, 1–1 Rokkodai, Nada, Kobe 657–8501, Japan

2 Department of Engineering and System Science, National Tsing Hua University, Hsinchu 30013, Taiwan

3 Engineering Biology Research Center, Kobe University, 1–1 Rokkodai, Nada, Kobe 657–8501, Japan

4 Department of Chemical Science and Engineering, Graduate School of Engineering, Kobe University, 1–1 Rokkodai, Nada, Kobe 657–8501, Japan

5 Center for Sustainable Resource Science, RIKEN, 1–7–22 Suehiro, Tsurumi, Yokohama 230–0045, Japan

^§^Current affiliation: Bacchus Bio innovation Co., Ltd.

†Equally contributed authors

*Correspondence to JI ([junjun@port.kobe-u.ac.jp](mailto:junjun@port.kobe-u.ac.jp))

*Correspondence to HYW ([hy.wang@gapp.nthu.edu.tw](mailto:hy.wang@gapp.nthu.edu.tw))

**Fig. S1. Schematic illustration of the batch culture method in a small shake flask used in this study.**


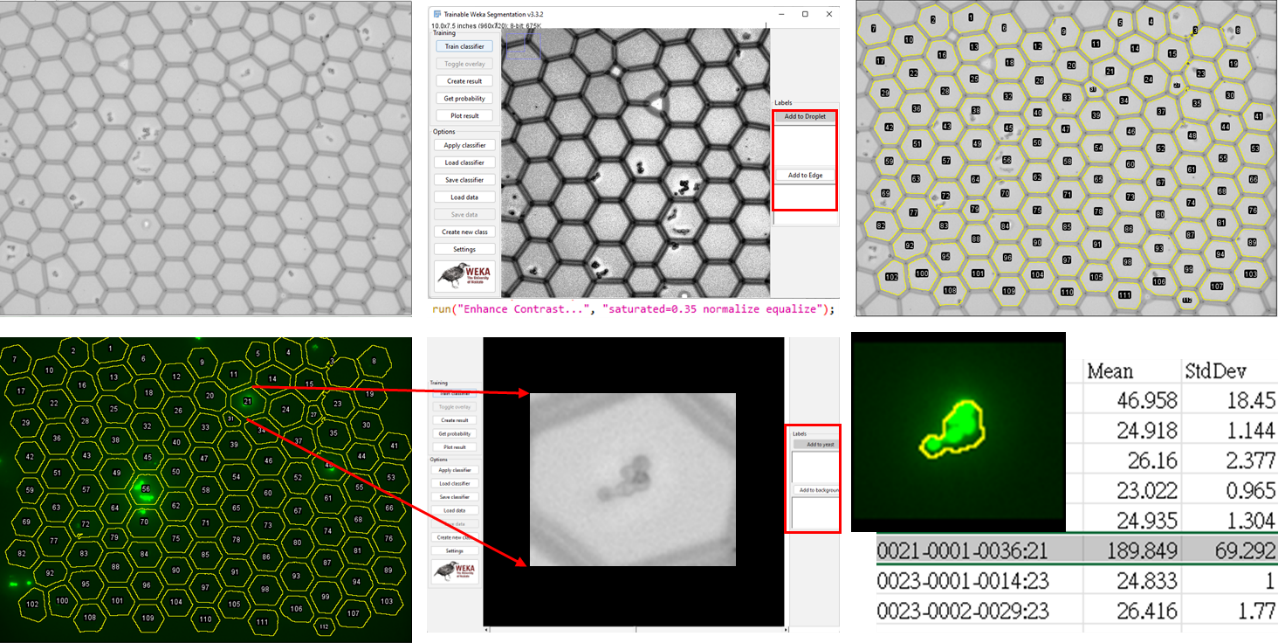


**Fig. S2. Custom macro incorporating machine learning techniques to identify droplets encapsulating yeast cells.** We used the “Trainable Weka segmentation” of the Fiji software for image processing in two steps: automatic single droplet image extraction and individual cell classification from the droplets. The learning method involved training the algorithm to recognize cell and droplet features to complete these steps. This approach is crucial for efficient data analysis, ensuring accuracy and speed.

**Fig. S3.** **Fluorescence-based, secreted Ang II peptide-stimulated AGTR1 signaling detection using engineered yeast in batch cultures.** **(A)** Single colonies were picked up and cultured in SD medium in test tubes. The cell cultures were then inoculated into SDM71 medium in flasks, and cultured at 30°C, shaken at 150 rpm. We observed the yeast cells under a fluorescence microscope and measured the fluorescence intensity using a flow cytometer. **(B)** Fluorescence images of yeast cells in batch cultures at 9 h of culture. **(C)** Time course of GFP fluorescence intensities in the P2 region of yeast cells (Additional File 1: Fig. S4A; fluorescent population) under batch-culture conditions. Approximately 10,000 cells were analyzed using flow cytometry. The error bars represent the mean ± SD of three independent experiments. N.D. = not detected.

**Fig. S4. Setting of the P2 and P2′ regions in batch and droplet cultures, respectively.** The engineered yeast cells (Mock and Ang II; Table 1) were used to determine the gate regions. **(A)** Histogram plots of the GFP fluorescence intensities of yeast cells cultured in batch flasks. Three independent yeast colonies were grown in flasks for 9 h, and approximately 10,000 cells were analyzed by flow cytometry. The P2 region was set to exclude non-fluorescent cells and include only fluorescent cells in the histogram plots of flow cytometry (P2% total in mock cells <1%). **(B)** Histogram plots of the GFP gray values of yeast cells cultured in single-cell microdroplets. The yeast cells were grown in W/O droplets for 9 h and observed under a fluorescence microscope. The gray values of >100 samples of yeast-incorporating droplets were measured by machine-learning-based image processing, as shown in Fig. 2B. The P2′ region was set to exclude non-fluorescent cells and include only fluorescent cells in the histogram plot of microscopy-based image processing (P2′% total in mock cells <1%).

**Fig. S5. Number of yeast cells encapsulated in a single droplet before cultivation (including empty droplets).**

**Fig. S6. Fluorescence-based detection of secreted angiotensin analog peptide-stimulated AGTR1 signaling using engineered yeasts in batch cultures.** GFP fluorescence intensities in the P2 region of yeast cells (Fig. S4A) in batch cultures at 9 h of culture. The error bars represent the mean ± SD of three independent experiments. Culture and analyses were performed using the procedures described in Fig. S3.

N.D. = not detected
